# GAAP: Genome-organization-framework-Assisted Assembly Pipeline for prokaryotic genomes

**DOI:** 10.1186/s12864-016-3267-0

**Published:** 2017-01-25

**Authors:** Lina Yuan, Yang Yu, Yanmin Zhu, Yulai Li, Changqing Li, Rujiao Li, Qin Ma, Gilman Kit-Hang Siu, Jun Yu, Taijiao Jiang, Jingfa Xiao, Yu Kang

**Affiliations:** 1 CAS Key Laboratory of Genome Sciences and Information, Beijing Institute of Genomics, Chinese Academy of Sciences, Beijing, 100101 China; 2 Center for Systems Medicine, Institute of Basic Medical Sciences, Chinese Academy of Medical Sciences & Peking Union Medical College, Beijing, 100005 China; 3 Suzhou Institute of Systems Medicine, Suzhou, 215123 China; 4 School of Life Sciences, Liaoning University, Shenyang, 110036 China; 5 Beijing SpeedyCloud Technologies Co., Ltd., Beijing, 110036 China; 6 Department of Otolaryngology, Beijing Geriatric Hospital, Beijing, 100095 China; 7 Department of Agronomy, Horticulture, and Plant Science, South Dakota State University, Brookings, SD 57007 USA; 8 Department of Health Technology and Informatics, The Hong Kong Polytechnic University, Hong Kong, China

**Keywords:** Core-gene-defined Genome Organizational Framework (cGOF), Scaffolding, Rearrangement, Prokaryotic genome

## Abstract

**Background:**

Next-generation sequencing (NGS) technologies have greatly promoted the genomic study of prokaryotes. However, highly fragmented assemblies due to short reads from NGS are still a limiting factor in gaining insights into the genome biology. Reference-assisted tools are promising in genome assembly, but tend to result in false assembly when the assigned reference has extensive rearrangements.

**Results:**

Herein, we present GAAP, a genome assembly pipeline for scaffolding based on core-gene-defined Genome Organizational Framework (cGOF) described in our previous study. Instead of assigning references, we use the multiple-reference-derived cGOFs as indexes to assist in order and orientation of the scaffolds and build a skeleton structure, and then use read pairs to extend scaffolds, called local scaffolding, and distinguish between true and chimeric adjacencies in the scaffolds. In our performance tests using both empirical and simulated data of 15 genomes in six species with diverse genome size, complexity, and all three categories of cGOFs, GAAP outcompetes or achieves comparable results when compared to three other reference-assisted programs, AlignGraph, Ragout and MeDuSa.

**Conclusions:**

GAAP uses both cGOF and pair-end reads to create assemblies in genomic scale, and performs better than the currently available reference-assisted assembly tools as it recovers more assemblies and makes fewer false locations, especially for species with extensive rearranged genomes. Our method is a promising solution for reconstruction of genome sequence from short reads of NGS.

**Electronic supplementary material:**

The online version of this article (doi:10.1186/s12864-016-3267-0) contains supplementary material, which is available to authorized users.

## Background

Next generation sequencing (NGS) technologies greatly promote genomic research of prokaryotes, generating tens of thousands of prokaryotic genome sequences in recent years. It is cost-effective and produces reliable data of high quality owing to high coverage. However, to achieve complete genome sequences of prokaryotes, the process of assembly and scaffolding are necessary, but always leave unordered assemblies and gaps due to short read length. Efficient and reliable scaffolding is a hurdle to investigate the regulatory and evolutionary profile based on linear and even high-dimensional genomic structure of microbial organisms [1–5].

Algorithms of *de novo* scaffolding, often build-in assembly software, such as SOAPdenovo [6], ABySS [7], and Velvet [8], rely on connections by pair-end (PE) reads and the length of insert size. Their performances are dramatically influenced by the length and abundance of repetitive regions of the target genome, such as ribosomal operons, transposases, and IS, which, if longer than insert size, are always undistinguishable. These repetitive regions cause conflicts as PE reads link them to non-unique contigs, and finally leaves the assemblies as fragmented draft. Therefore, more information is needed to orientate and order the disconnected scaffolds and contigs.

Since the prokaryotic genomes often follow phylogenetic relationship, reference genomes would be helpful in such case, and therefore, the reference-assisted algorithms emerge [9–13]. Among them, typically, AlignGraph extends and links contigs with PE reads under the guidance of a reference genome of a closely related organism; Ragout uses one or multiple references along with the phylogenetic relationship to order the contigs. Species with conserved genomic structure fit well with these algorithms. However, the flexibility of genome structures is elusive, different species might have various genomic complexity [14]*.* Even draw support from phylogeny, rearrangement might be so intensive that closely related strains may have distinct genome organization, whereas isolates with the same genomic organization may present in remotely relative strains [14–16]. These studies suggest that genomic rearrangement can be independent of phylogenetic relationship of genomic content, which would cause systemic errors if the algorithm relies deeply on phylogeny to select references for scaffolding.

Although prokaryotic genomes can be extensively rearranged within a species, core genes are more stable in term of position than dispensable genes in genomic scale. The core genome of species, defined as cGOF (core-gene-defined Genome Organizational Framework) in our previous study, constitutes of those genes that are vertically inherited with conserved order, i.e. keep synteny in generations, in the range of whole genome or a large segment [16]. On the contrary, the other genes in a genome, i.e. dispensable genes are subject to horizontal gene transfer, and often change their positions in genome. The discrepancy of position conservation between core and dispensable genes proposes a scaffolding algorithm that orders original assemblies according to the cGOF. In this way, we have finished ten self-sequenced genomes of *E. coli* isolates. In these strains, all neighboring relationship of scaffolds and contigs we predicted based on cGOF were verified using PCR if not strongly supported by PE reads [16].

Here, we implement the algorithm based on cGOF creating a program GAAP (cGOF-assisted assembly pipeline). Rather than starting with a selection of reference genome(s), we use pangenomic method to extract the order-conserved cGOF genes for scaffolding and supplement with PE reads to extend the connections between original assemblies and close gaps. Hereto, a draft of a few scaffolds that counts less than the cGOF segments of the species can be obtained. Further, GAAP suggests a permutation of the scaffolds according to the most prevalent and conflict-free segment permutations in the references, and thus achieve a circular assembly. The construction of the pseudo-genome can be further validated by PCR if the strain is available. As the biological feature of genome rearrangement is species-specific, prokaryotic species can be classified into three categories according to their cGOF patterns: single-segment, symmetric, and asymmetric multiple-segment cGOF [16]. Here, we compare GAAP to three other reference-assisted programs, Ragout, MeDuSa and AlignGraph, and demonstrate that GAAP achieves the paralleled performance using both empirical and simulated data in species with diverse genome size, complexity, and all the three categories of cGOF.

## Methods

### Test data-set

Fifteen genomes in six species of various genome size, complexity, and cGOF pattern are used for performance test. All test genomes have complete genome sequences and PE reads data. The genomes are downloaded from NCBI FTP with accession numbers as follows: NC_003923 (MW2) and NC_017338 (JKD6159) for *Staphylococcus aureus*, NC_000913 (MG1655) for *Escherichia coli*, NC_018936 (A20) and NC_020540 (M1 476) for *Streptococcus pyogenes*, NC_011333 (G27), NC_020509 (OK310) and NC_017359 (Sat464) for *Helicobacter pylori*, CP002640 (SS12), CP002644 (D12), CP002651 (ST1), CP002641 (D9) and CP002570 (A7) for *Streptococcus suis*, and NZ_AKGH01000001 (H1 chr 1) for *Vibrio cholera*. Except for the five S. *suis* strains [17, 18] and the *E. coli* strain (NCBI SRA/SRR001665), for which real reads data are available, the reads of all the other strains are simulated by using wgsim package from SAMtools [19] with base error rate of 0.02, 2 × 100 bp and physical coverage no less than 100×. All above reads of the genomes are *de novo* assembled by using SOAPdenovo to generate original assemblies [6].

### The frame of GAAP

**Fig. 1:**
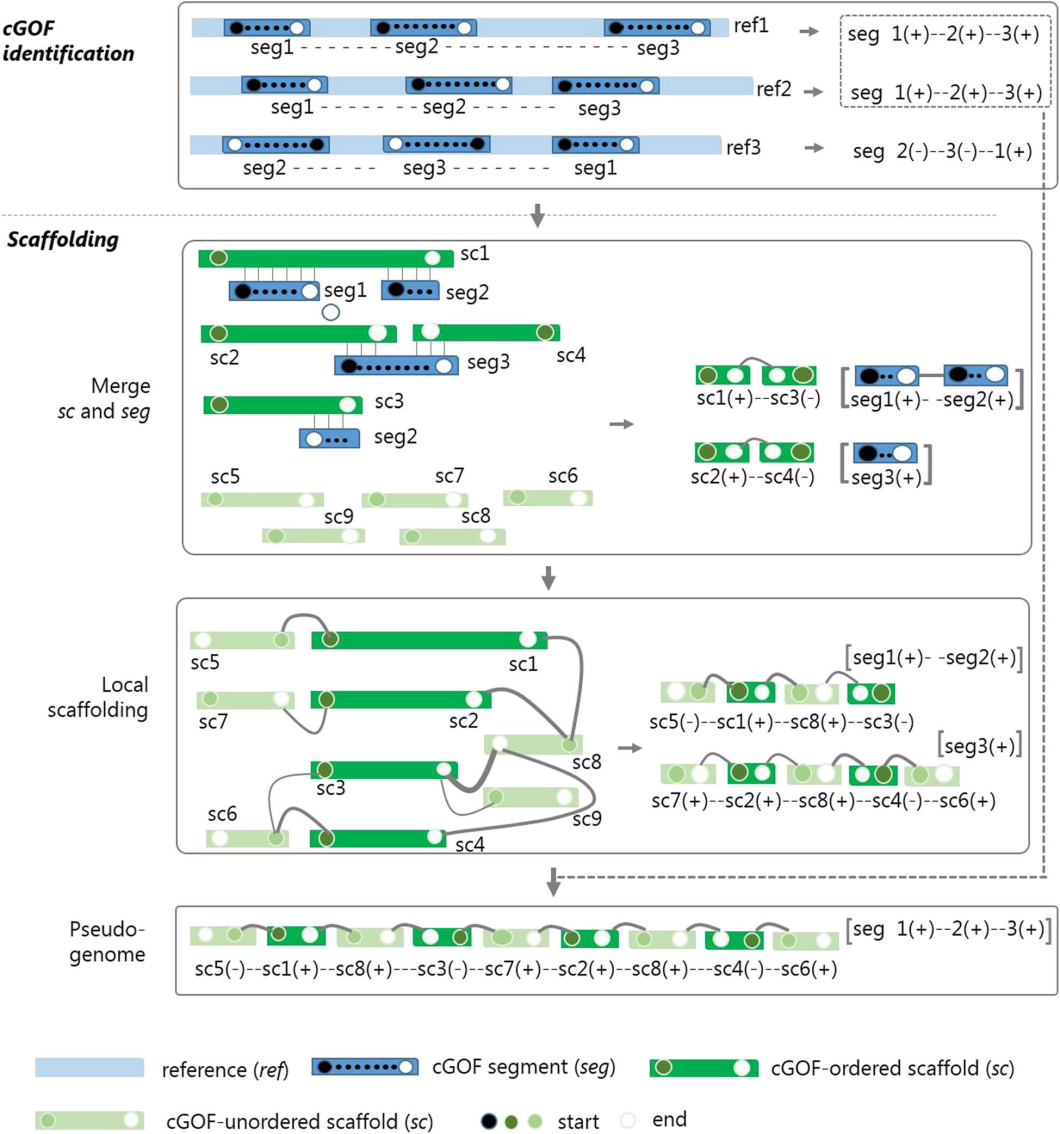
The schematic framework of GAAP. seg, segment of cGOF. ref, reference; sc, scaffold/contig (assemblies). Head (filled circle) and tail (empty circle) vertices of the syntenic seg in each reference are sequentially connected with a dashed line indicating the seg permutation (order and orientation). The sc are indexed with seg and merged into ordered sc “strings”. The graph in the local scaffolding of ordered sc is built by connecting seg-ordered sc and unordered sc, where the PE links are higher than a certain cut-off. The line widths indicate the link count. Pseudo-genome of draft-quality assembly is constructed by combining the indexed scaffolds and the closest relevant seg permutaion of references

GAAP is schematized into two major steps: cGOF identification and scaffolding (Fig. 1). Before start, we recommend to use PGAP (pan-genomes analysis pipeline) [20] to generate the core gene cluster from a set of complete reference genomes. cGOF identification is designed to identify the syntenic cGOF segments from genomic positions information of each core gene ortholog. It takes the core gene cluster and their genomic position in each reference genome as input, and outputs cGOF segments of the species and their permutations in reference genomes. The second step, scaffolding, is to construct a pseudo or draft genome for each target by aligning the original assemblies to cGOF segments and then filling gaps by PE reads mapping. It takes the output of the first step (cGOF of the species), PE reads, and original assemblies as input. Additionally, if a draft with a few scaffold “strings” comes out, the GAAP suggests their permutation follows the conflict-free and most prevalent one of the references.

### cGOF segment identification

The process of cGOF segment identification has been described in Kang et al. [16], where only large cGOF segments are counted. To recover more original assemblies, we set a cutoff for segment length as the minimal of two consecutive core genes. The shorter the cutoff, more core genes will be identified in cGOF segments (but possibly less reliable), and more original assemblies will be recovered. The process of cGOF segment identification is as following (see Fig. 1 “*cGOF identification”*): we first sort the single-copy core genes according to their order in each reference genome, and then use a self-developed iteration algorithm to obtain all syntenic segments, where the single-copy core genes keep stable linear order. Here, we strictly define cGOF segments as subsequences composed of cGOF genes in consecutive order, and record the permutation of all segments of each reference genome.

### Scaffolding

Scaffolding is implemented by ordering and orientating the original assemblies according to the indexes of cGOF genes. Additionally, PE reads are used to link the neighboring assemblies, called local scaffolding. Further, a pseudo-genome is output if the cGOF segments are permutated as the most prevalent references that do not conflict with indexes of the assemblies.

#### Indexing the original assemblies

The original assemblies are searched for cGOF genes by using BLAT, and then indexed by cGOF genes for order and orientation of the assemblies. Herein, one segment might span multiple assemblies, vice versa, and the mutual overbridges between them assemble the original assemblies into a few scaffold “strings” (Fig. 1 *“Merge sc and seg”* and Additional file 1: Figure S1). The original assemblies that do not contain any cGOF genes or not uniquely mapped are not joined in.

#### Local scaffolding

In contrast to scaffolding in the genomic scale, local scaffolding is to use PE reads to: 1) confirm the neighboring relationship of original assemblies predicted based on cGOF, and 2) recover assemblies which were not joined in and often represent repetitive regions. PE link pairs between the assemblies are screened to ensure the count greater than a cut-off (default 5) to exclude connections caused by systematic errors. For each pair of assemblies, sc_i_ and sc_j_, there exists four types of connection between them, (i) head-to-head, [sc_i_(−),sc_j_(+)] or [sc_j_(−),sc_i_(+)];(ii)head-to-tail, [sc_i_(−),sc_j_(−)] or [sc_j_(−),sc_i_(−)]; (iii) tail-to-head, [sc_i_(+),sc_j_(+)] or [sc_j_(+),sc_i_(+)]; (iv) tail-to-tail, [sc_i_(+),sc_j_ (−)] or [sc_j_(+),sc_i_(−)]; where positive and negative signs indicate the orientation of assemblies. The graph of local scaffolding is constructed based on a complete evaluation of the confidence of pairwise connection, which is done by combining the permutation of assemblies indexed by the cGOF, and the count of PE reads that support the link which might not be effectively used to join contigs solely based on read pairs. The graph consists of head and tail vertices that represent the head and tail of each assembly, and their connected edges. Each edge has a weight confidence in the range of 0–1 that indicates how confident the connection of the two vertices is. For each edge (i,j), by combining the permutation and pair links between two vertices i and j, the weighted confidence c(i.j) is defined as:

$$ \mathrm{c}\left(\mathrm{i},\mathrm{j}\right)=a*\mathrm{permutation}\left(\mathrm{i},\mathrm{j}\right)+\left(1-a\right)*\mathrm{link}\left(\mathrm{i},\mathrm{j}\right), $$

where *a* controls the relative contribution of permutation and pair links. Confidence of edges between the head and tail vertices of one assembly, and edges representing connections consistent with the cGOF order, are designed as one, while those conflict with the cGOF order are always zero. Other vertices are confidently connected only when their weights are greater than zero or got the highest value when more than one edges compete for one vertice. Finally, based on the order and orientation that are inferred from the chains of graph, GAAP concatenates the assemblies into a pseudo genome or a draft of a few scaffolds “strings”. See Fig. 1 “*local scaffolding”* and Additional file 2: Figure S2 for demonstration of examples. In this process, original assemblies that were not indexed by cGOF can be recovered, and even reused when link to multiple other assemblies.

#### Output a pseudo-genome

For all the permutations of cGOF segments in references, those conflicting with original assemblies are removed at first, and then the remaining permutations are sorted according to their prevalence in reference genomes. Finally, the most prevalent one is chosen to guide the scaffold “strings” into a pseudo-genome. If the indexed assemblies conflict with all the permutations in the references, there will be no output of pseudo-genome, which indicates a novel arrangement pattern in the target genome.

## Results and Discussion

We evaluated GAAP performance against three other reference-assisted tools, Ragout, MeDuSa and AlignGraph by using the same reference sets (see Additional file 3: Additional Text File). For Ragout, we run three iterations with minimum synteny block sizes (5000, 500, 100) with refinement, and for GAAP, MeDuSa and AlignGraph, no extra settings are set except the default parameters. Their performances in term of errors and N50 metrics was evaluated by GAGE [21]. Since GAGE reports only events, i.e. number of breaks in the alignment, we also tallied coverage, i.e. length of recovered and relocated assemblies for supplement. Here, we define coverage as the length ratio of the recovered/relocated assemblies to the reference complete genome in term of percentage, and errors as number of breaks in alignment, including indels, inversions and relocations tallied by GAGE.

Since genomic rearrangement is very challenging to reference-assisted assemblers, we first used three genomes of species *S. pyogenes*, *H.pylori* and *V.cholerae*, which are known for frequent rearrangement. Compared with GAAP, Ragout and MeDuSa, AlignGragh generated a draft-quality assembly of much lower coverage and more final scaffolds, while GAAP, Ragout and MeDuSa produced one final scaffold for each of the testing genome, and exhibited comparable coverage and errors (Table 1). Although AlignGraph produced less errors, it might be influenced by its less aggressive algorithm which recovered less assemblies.

**Table 1:** Performance of reference-assisted assembly tools

	*S. pyogenes* A20	*H. pylori* G27	*V. cholerae* H1 chr1
Ref complete genome, Mb			
	1.85	1.67	3.08
Original assemblies			
Number (>300 bp)	38	40	41
N50, kb	124	134	236
Final scaffolds			
GAAP	1	1	1
AlignGraph	11	7	15
Ragout	1	1	1
MeDuSa	1	1	1
Coverage, recovered % (falsely located %)			
GAAP	98.65 (0.15)	98.57 (0.06)	97.85 (0)
AlignGraph	81.93 (8.78)	61.75 (0)	79.13 (0)
Ragout	97.35 (17.7)	98.28 (0.02)	98.05 (0)
MeDuSa	98.2 (19.7)	98.95 (0.10)	98.19 (11.15)
Errors			
GAAP	19	5	8
AlignGraph	3	2	1
Ragout	6	5	5
MeDuSa	9	11	14
Corrected N50, kb			
GAAP	273	277	2,026
AlignGraph	124	138	351
Ragout	103,6	1,121	1,323
MeDuSa	252	207	245

To further discern the performance of GAAP, Ragout and MeDuSa, we recruited 12 other genomes in five distinct bacterial species characterized by different cGOF patterns as single-segment, symmetric, and asymmetric multi-segment, as well as variable genome sizes and complexity.

Firstly, for genomes with single-segment cGOF, we took two genomes of *S. aureus* for test, which are 2.8 Mb in length and exhibit stringent synteny in most core genes. The three methods all achieved single pseudo-genome with coverage of over 98%. For species of this cGOF pattern, most reference-assisted assemblers can achieve high-quality assembly with high coverage and accuracy. GAAP recovered more assemblies, and slightly less errors and longer N50 than the other two (Table 2).

**Table 2:** Performance on species of single-segment cGOF

	*S.aureus* MW2	*S.aureus* JKD6159
Reference genome, Mb		
	2.82	2.81
Original assemblies		
number (>300 bp)	12	26
N50 (kp)	1,416	262
Coverage, recovered % (falsely located %)		
GAAP	99.0 (0)	98.75 (0)
MeDuSa	99.06 (17.6)	99.2 (3.01)
Ragout	99.05 (0)	98.62 (0)
Errors		
GAAP	9	3
MeDuSa	11	6
Ragout	10	4
Corrected N50, kb		
GAAP	1,519	2,276
MeDuSa	499	637
Ragout	1,534	1,757

Next, we turned to species with symmetrical cGOF, and took six genomes in *S.pyogenes* and *S.suis* besides the two in Table 1. Genomes with symmetric cGOF contain two or four cGOF segments which often rearrange symmetrically around the Ori-Ter axis of replication. GAAP, Ragout and MeDuSa produced single pseudo-genome for each target, but with some errors due to the more complex genome organization. Since GAAP uses stable cGOF indexes to order the assemblies instead of specific reference sequences which might be distinct from the target, we suppose the original assembly is correct and keep all the variations including SNPs, indels and structure variations in the final assembly, while Ragout and MeDuSa align assemblies directly against the reference genomes and refine the assemblies. Therefore, GAAP did not show advantage in errors reported by GAGE even when it recovered more assemblies with fewer false location (Table 3).

**Table 3:** Performance on species with symmetrical cGOF

	*S.pyogenes* M1 476	*S.suis* SS12	*S.suis* A7	*S.suis* D12	*S.suis* ST1	*S.suis* D9
Reference genome, Mb						
	1.86	2.18	2.12	2.26	2.12	2.14
Original scaffolds						
Number (>300 bp)	27	32	41	74	86	67
N50, kp	123	170	166	73	45	71
Coverage, recovered % (falsely located %)						
GAAP	97.17 (8.78)	97.28 (1.53)	94.53 (1.56)	94.04 (0)	98.16 (0.69)	96.10 (0)
MeDuSa	98.99 (9.13)	98.70 (0.41)	98.61 (0.04)	91.37 (37.12)	94.50 (37.79)	95.10 (0.20)
Ragout	97.09 (8.78)	95.76 (38.08)	93.34 (1.56)	94.90 (3.05)	94.36 (6.12)	95.60 (38.41)
Errors						
GAAP	6	14	17	29	20	24
MeDuSa	9	7	10	26	16	13
Ragout	5	12	7	16	12	12
Corrected N50, kb						
GAAP	1292	478	258	284	304	244
MeDuSa	964	1,433	311	142	208	368
Ragout	1,330	414	1,212	338	654	398

In species with symmetric cGOF, although genome structure varies somewhat, core genes still keep synteny in long genomic ranges, and the left and right arm segments exchange their position systemically. Large misjoined fragments occur only when rare rearrangement are present in the target genomes and the rearranged fragments cannot be joined to neighboring segment by overbridged assemblies. Another feature of species with symmetric cGOF is that genome organization, or permutation of cGOF segment, can be reversely rearranged around the Ori-Ter axis, and independent of phylogenetic relationship. Since phylogenetics is what Ragout refers to select reference genomes, it will occasionally misleads assembly, whereas GAAP, independent of phylogenetic relationship and specific references, exhibit apparent outperformance in coverage in this suite of cases.

Finally, we recruited four genomes in species *E. coli* and *H. pylori* with asymmetric cGOF. For the strains *E.coli* MG1655, both empirical and simulated PE data were tested. The results showed that the three methods achieved comparable results (Table 4). Although GAAP had more errors number in some cases, it still exhibited superior coverage, especially the falsely located assemblies. In species with asymmetric cGOF, although there are much more segments that are extensively rearranged, GAAP also perform well with the support from cGOF indexes and the prevalence information of segment permutation in reference genomes. In contrast to symmetric cGOF species, genome rearrangement in species with asymmetric cGOF is largely correlated with phylogeny, and thus Ragout performed almost as well as GAAP in most cases. Although GAAP does not depend on phylogeny, closely related reference genomes will provide more relevant information on core gene set, composite segments, their permutation and prevalence, and thus provide more accurate guidance for assembly. For species of this cGOF pattern, if there is empirical evidence of the references and the target, selection of closely related reference genome will improve performance of these methods.

**Table 4:** Performance on species of asymmetrical cGOF

	*E.coli* MG1655^a^	*E.coli* MG1655^b^	*H.pylori* OK310	*H.pylori* Sat464
Reference genome, Mb				
	4.71	4.71	1.61	1.58
Original assemblies				
number (>300 bp)	105	85	29	39
N50, kb	105	176	165	142
Coverage, recovered % (falsely located %)				
GAAP	91.89 (0)	95.29 (0)	98.91 (0)	89.30 (5.23)
MeDuSa	97.25 (0.96)	97.89 (34.82)	99.29 (10.13)	89.61 (16.01)
Ragout	96.19 (0.05)	95.45% (0)	98.48 (4.76)	89.08 (5.43)
Errors				
GAAP	20	33	13	14
MeDuSa	29	13	9	7
Ragout	21	21	25	9
Corrected N50, kb				
GAAP	411	313	1292	190
MeDuSa	268	308	964	348
Ragout	689	689	1330	348

^a^Empirical PE reads data downloaded from NCBI SRA (SRR001665)

^b^Simulated PE reads data

## Conclusion

We have presented a new algorithm for generating pseudo-genomes of prokaryotes based on the concept of cGOF, that is, the syntenic segments of core genes of a species. We implement this algorithm creating program GAAP, which is to our knowledge the first reference-assisted scaffold program that explicitly models the biological feature of cGOF and takes advantage of the reliability of core genes on their position information. We compared GAAP to three other recently presented programs, and demonstrated that GAAP exhibited no inferiority on both empirical and simulated data and diverse suites of test cases, even when the other three also stem from taking advantage of reference refinement.

As genomic data of prokaryotes have been rapidly accumulated since the launch of NGS, it is no longer an obstacle to prepare enough references for GAAP (usually over ten). Ideally, obtaining complete genomes of all strains is promising for large pangenomic studies, and GAAP provides an economic and rapid solution with high accuracy for species with various genome size, complexity, and cGOF pattern. As more genomic data are accumulated and there are sufficient alternatives, a possible improvement of GAAP is to optimize the selection of references, under the rationale that references with broader diversity and closer phylogenetic relevance can give a more accurate prediction of the target genome.

## Availability and requirements

**Project name**: GAAP

**Project home page**: http://gaap.big.ac.cn

**Operating system(s)**: Platform-independent

**Programming language**: Python

**Other requirements**: Python 2.7 or higher

**License**: GNU GPL

**Availability**: GAAP, including source code, documentation, and examples, is freely available for non-commercial use with no restrictions at http://gaap.big.ac.cn.

## Additional files

Additional file 1: Figure S1. Scheme of merging of scaffolds and cGOF segments. Each column and row indicate a scaffold and cGOF segment from start (top/left) to end (bottom/right) respectively. cGOF segments are consisted of cGOF genes in stable order. By sequence alignment, scaffolds will be indexed by cGOF genes, and ordered and orientated into scaffold “strings”, vice versa. The mutual overbridges between them assemble the original assemblies into large scaffolds, and construct the cGOF skeleton of the target strain. (JPEG 234 kb)
Additional file 2: Figure S2. Scenario of conflicting links between contigs. Black short lines indicate scaffolds and contigs with a,b,c,d ordered and r, s unordered ones. The widths of curve lines indicate the link count. (JPEG 31 kb)
Additional file 3: Additional Text File. The sets of reference genomes for GAAP and Ragout. (DOCX 18 kb)

## Abbreviations

cGOF: Core-gene-defined Genome Organizational Framework
GAAP: Genome-organization-framework-Assisted Assembly Pipeline
NGS: Next generation sequencing
PE: Paired-end
PGAP: Pan-genomes analysis pipeline
