# Supplementary figures and images for: GAAP: Genome-organization-framework-Assisted Assembly Pipeline for prokaryotic genomes
Source: BMC Genomics. 2017 Jan 25;18(Suppl 1):952. doi: 10.1186/s12864-016-3267-0 (PMC5310280; doi:10.1186/s12864-016-3267-0)

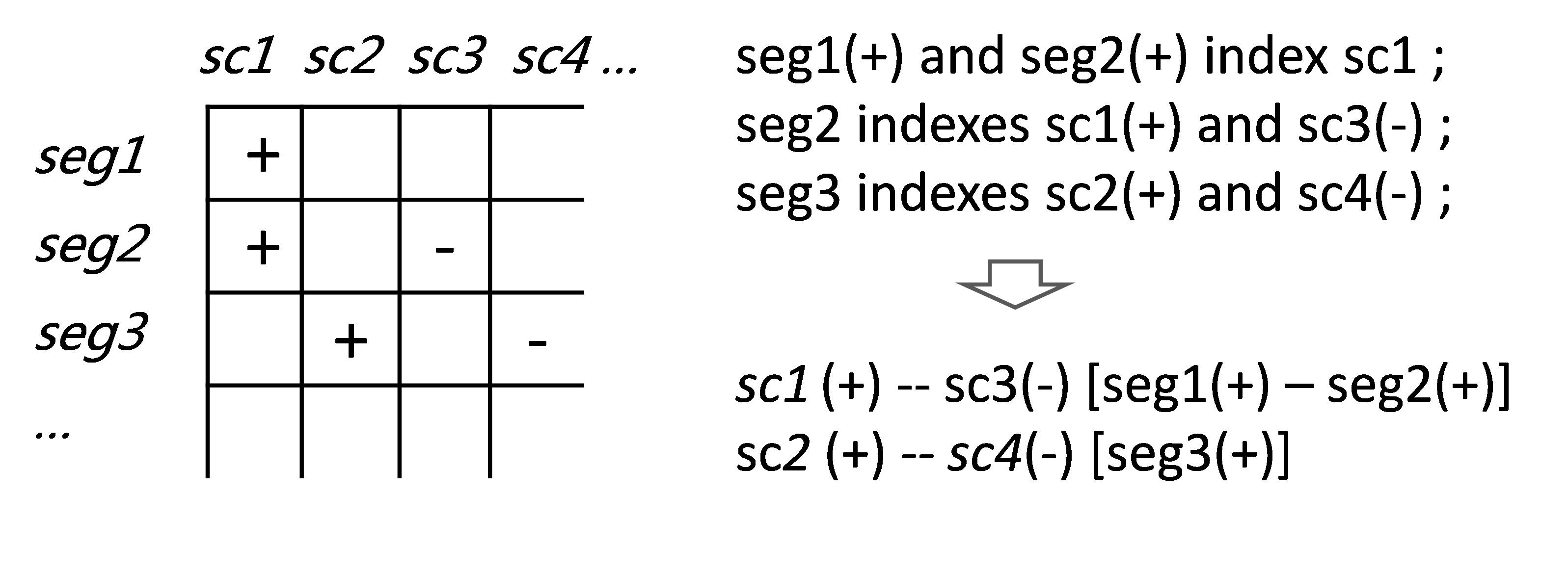

Supplement: Additional file 1: Figure S1. — Scheme of merging of scaffolds and cGOF segments. Each column and row indicate a scaffold and cGOF segment from start (top/left) to end (bottom/right) respectively. cGOF segments are consisted of cGOF genes in stable order. By sequence alignment, scaffolds will be indexed by cGOF genes, and ordered and orientated into scaffold “strings”, vice versa. The mutual overbridges between them assemble the original assemblies into large scaffolds, and construct the cGOF skeleton of the target strain. (JPEG 234 kb) [file 12864_2016_3267_MOESM1_ESM.jpeg]

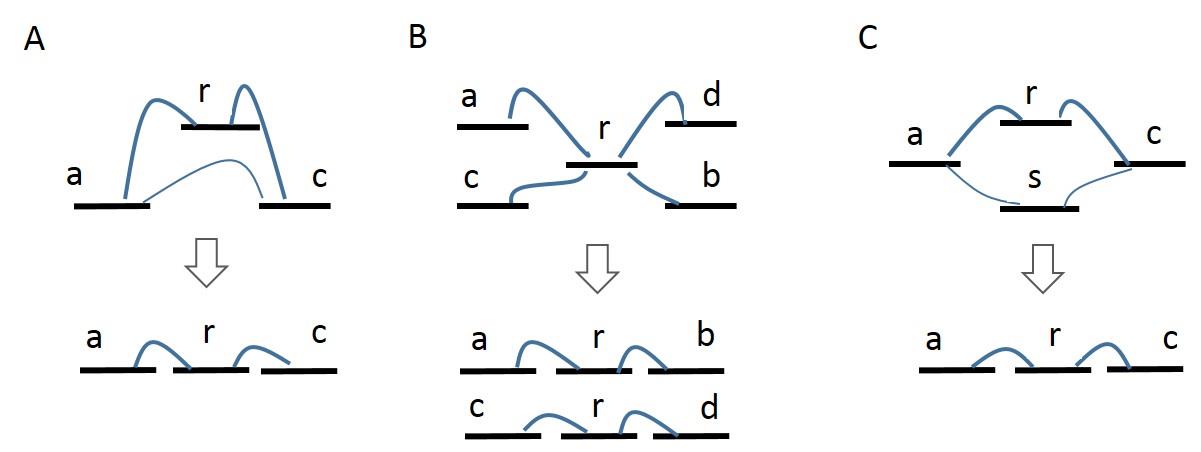

Supplement: Additional file 2: Figure S2. — Scenario of conflicting links between contigs. Black short lines indicate scaffolds and contigs with a,b,c,d ordered and r, s unordered ones. The widths of curve lines indicate the link count. (JPEG 31 kb) [file 12864_2016_3267_MOESM2_ESM.jpeg]
