# Supplementary material for: GAAP: Genome-organization-framework-Assisted Assembly Pipeline for prokaryotic genomes
Source: BMC Genomics. 2017 Jan 25;18(Suppl 1):952. doi: 10.1186/s12864-016-3267-0 (PMC5310280; doi:10.1186/s12864-016-3267-0)
Supplement: Additional file 3: — Additional Text File. The sets of reference genomes for GAAP and Ragout. (DOCX 18 kb) [file 12864_2016_3267_MOESM3_ESM.docx]

**Additional Text File**

The sets of reference genomes for GAAP,MeDuSa and Ragout.

The reference set of *S. aureus* contained nine other finished genomes (accession number NC_002745, NC_017331, NC_020568, NC_009487, NC_007622, NC_016941, NC_016928, NC_002952, NC_017673, NC_002953, NC_007795, NC_007793). Phylogeny used in Ragout was ((((((NC_007795,NC_007793),NC_017331),(NC_002953, MW2)),NC_016928),((NC_007622, JKD6159),(NC_002745,(NC_009487,NC_020566)))),(NC_002952,NC_017673)).

The reference set of *S. pyogenes* contained eight reference genomes (NC_008021, NC_008024, NC_002737, NC_017040, NC_011375, NC_009332, NC_017596, NC_008023) .The phylogenetic trees used in Ragout was (NC_008021,(((((A20,NC_002737),M1476),(NC_008021,NC_008023)),NC_009332), (NC_011375,(NC_017040,(NC_008024,NC_017596)))).

The reference set of *S. suis* contained 12 genomes (NC_009442, NC_009443, NC_012924, NC_012925, NC_012926, NC_017617, NC_017618, NC_017619, NC_018526, NC_020526, NC_021213, NC_022665). The phylogenetic trees used in Ragout was (((((((((((CP000407,CP002465),A7),(AM946016,(CP000408,CP002640))),CP000837),CP003736),CP003922),FM252031),FM252032),SS12),ST1),(D12,(CP003993, (CP006246,D9))));

The reference set of *E. coli* consisted of 19 finished genome deposited in Genbank (accession number NC_011748, NC_008253, NC_013361, NC_013353, NC_012759, NC_013941, NC_004431, NC_010473, NC_011601, NC_009801, NC_011353, NC_011745, NC_002655, NC_009800, NC_011741, NC_011750, NC_011742, NC_011415, and NC_011751). The phylogenetic tree used in Ragout was ((((((((((MG1655,NC_010473),NC_012759),NC_013361),(NC_013353,NC_002655)),NC_009801),((NC_011748,NC_011415),(NC_009800,NC_011741))),(NC_013941,NC_011353)),NC_011750),(NC_008253,NC_011745)),(NC_011751,(NC_004431,(NC_011601,NC_011742))));

The reference set of *H. pylori* consisted of 16 genomes (accession number NC_000915, NC_017378, NC_017375, NC_017379, NC_017926, NC_017355, NC_010698, NC_000921, NC_017063, NC_017372, NC_017361, NC_008086, NC_012973, NC_014555, NC_017354, and NC_017382); The phylogenetic tree used in Ragout was (((((((((NC_017378,NC_017379),NC_010698),NC_017355),Sat464),(NC_017926,(OK310,NC_017375))),NC_017372),(NC_000915,(NC_017361,(NC_000921,NC_017063)))),NC_008086),G27).

The reference set of *V.cholera* consisted of seven genomes (accession number NC_0025056, NC_0094576, NC_0125780, NC_0125823, NC_0126687, NC_0164456, NC_0169445, and NC_0172709). The phylogenetic tree used in Ragout was (((((NC_0164456,NC_0169445),NC_0126687),(NC_0025056,NC_0125780)),(NC_0094576,NC_0125823)), H1);
